# Role of Perioperative Monitoring in Diagnosis of Massive Intraoperative Cardiopulmonary Embolism

**DOI:** 10.15171/jcvtr.2014.002

**Published:** 2014-09-30

**Authors:** Ognjen Visnjevac, Leili Pourafkari, Nader D. Nader

**Affiliations:** ^1^Departments of Anesthesiology, Critical Care, and Surgery, University at Buffalo, Buffalo, New York, USA; ^2^Cardiovascular Research Center, Tabriz University of Medical Sciences, Tabriz, Iran

**Keywords:** Plumonary Embolism, Intraoperative Period, Echocardiography, Pulmonary Artery Catheter, End-Tidal Carbon Dioxide, Monitoring

## Abstract

Massive thrombotic intraoperative pulmonary emboli (IOPE) is rare but carries a great degree of morbidity and mortality. This is the first study to formally assess the utility of various tools for the diagnosis of these events and the impact of each tool on mortality. Due to both the infrequent occurrence of these events and the high mortality of massive IOPE, it was cost-prohibitive to prospectively randomize patients to study commonly used diagnostic tools. Hence, a descriptive review of all reported cases in the literature was performed. This review yielded 146 cases for past 4 decades. Following a careful review of these cases, the alerting monitor for the occurrence of IOPE was recorded. Furthermore, we recorded the confirming diagnostic tool and the outcome of these patients. We compared 4 monitoring tools: (1) end-tidal carbon dioxide; (2) central catheter pressures; (3) echocardiography; and (4) standard monitoring of vital signs. Pre-event use of transesophageal echocardiography had no survival benefit. End-tidal carbon dioxide changes as an alerting tool were associated with improved survival compared to changes in vital signs (P<0.0001). Signs of right heart strain were associated with greater mortality, but direct thrombus visualization was not. Echocardiography appears to be useful for diagnosis of massive IOPE. Compared with hemodynamic collapse, end-tidal carbon dioxide decline as the presenting sign of massive IOPE may be associated with a better prognosis because it may represent earlier detection of IOPE and allow for more time to intervene.

## Introduction

Massive thrombotic intraoperative pulmonary emboli (IOPE) remain rare events, but they hold a high degree of morbidity and mortality.^1-3^ While great strides have been made toward reducing the incidence of perioperative pulmonary embolism, there are no consensus guidelines for screening, diagnosis, or management of massive pulmonary emboli when they occur in the operating room.^4,5^ Various management strategies for massive IOPE have been reported, and they appear to favor aggressive intervention over supportive care,^2^ but this is the first descriptive study to systematically assess the survival benefit of several common intraoperative screening, monitoring, and diagnostic tools.

Due to both the infrequent occurrence of IOPE events and the high mortality associated with them, it would have been both cost-prohibitive and ethically challenging to prospectively study these events. A descriptive study design was employed to identify the monitoring, screening, and diagnostic tools used during reported cases of IOPE as well as those we encountered at affiliate hospitals, extensively supplemented with data from cases identified through literature review. As practices were not protocol-drive, the four studied diagnostic strategies were broadly and inclusively defined: (1) end-tidal carbon dioxide monitoring (EtCO2); (2) central catheter pressure monitoring (PAC); (3) transesophageal or transthoracic echocardiography (TEE); and (4) standard operative monitoring of vital signs (VS).

We hypothesized that all the alerting tools (EtCO2, VS, and PAC) would be useful screening tools, but would not yield an improvement in mortality. Furthermore, we hypothesized that direct visualization of thrombus by TEE would be the only clinical tool associated with a survival benefit as it might yield more rapid diagnosis and more directed therapy.

## Materials and methods

In order to assess the value of each clinical tool, it was of utmost importance to collect data on all available cases of IOPE. A case series of IOPE cases from affiliated hospitals was collected and extensively supplemented with data from relevant cases obtained by literature review. To identify the relevant literature, we screened articles for inclusion and exclusion criteria using ISI, EBSCO-CINAHL, PubMed and OVID (1946-2013) without limitation in date or language of publication. The search strategy was initiated using the words:* pulmonary embolism or thromboembolism,* in combination with *intraoperative, surgery, or anesthesia*.

To meet inclusion criteria, articles must have described cases in which the initial presenting signs and symptoms of a reported thrombotic-type massive pulmonary embolus (PE) occurred during the operative period. Massive emboli were broadly defined as those that were rapidly hemodynamically compromising. The criteria for diagnosis of pulmonary emboli were not preemptively or objectively defined as this study was designed to be inclusive and descriptive of recent clinical practices. Such practices (including diagnostic modalities) were expected to differ considerably as this study encompassed data from several decades, both nationally and internationally. Hence, diagnosis was left to the discretion of each clinician, and in some cases, was made post-mortem. Cases met exclusion criteria if they described non-thrombotic emboli, emboli occurring prior to or following the surgical procedure, or cases in which emboli did not result in hemodynamic compromise.

**Figure 1:**
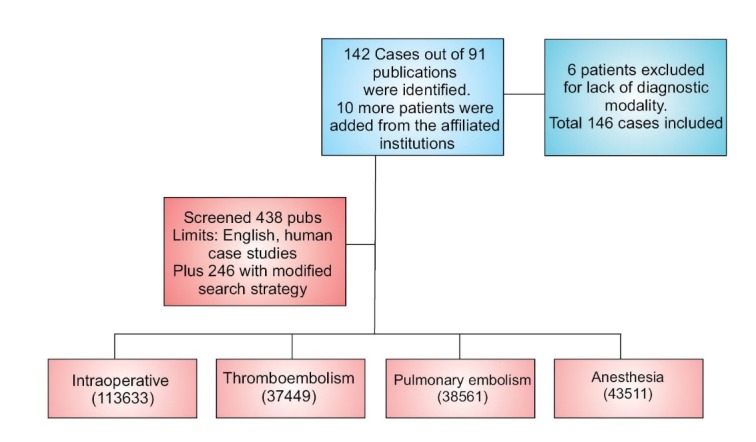
Flow chart depicting the search strategy with the keywords and the limits used to include cases in statistical analyses.

The initial literature search yielded 438 potential reports. Additional 246 articles including abstracts and conference proceedings were screened from the reference list of the searched publications. 152 cases met all criteria, but 6 of these cases (all from one report) were excluded as they merely reported the occurrence of massive PE’s resulting in intraoperative deaths, but did not report the diagnostic modalities or tools employed for any of the 6 cases. The authors of this report were not available for clarification. Among the 146 cases to be reviewed were 10 unpublished reports of cases of massive IOPE from hospitals affiliated with our University (Figure 1). For a complete list of case references, including the unpublished reports from our institution, see “Supplementary file 1”.

Data from cases identified for inclusion were used for two purposes: (1) to assess which modalities most frequently alerted practitioners of the massive IOPE event and the associated outcomes; and (2) to assess the diagnostic utility of echocardiography for massive IOPE, in reference to the primary outcome of in-hospital mortality. EtCO2 monitoring, PAC pressure monitoring, and changes in vital signs were included in the descriptive assessment of alerting tools used by clinicians. Specific echocardiographic findings were reported when available. In cases in which the authors did not report the use of a specific diagnostic modality, these data points were marked as missing, subsequently reducing relevant denominators for data analysis.

Additionally, all cases were identified by their year of publication, which was used to stratify cases on a per-decade basis. This was then used to identify changes in clinicians’ practices and diagnostic strategies over time, with reference to the primary outcome of in-hospital mortality.

### Statistical analyses and data collection

Data were entered into a Microsoft Excel worksheet, then transferred to a NCSS datasheet version 2007 (Salt Lake, UT) for statistical analysis. Univariate analyses were done using Chi-squared test. P values less than 0.05 were considered statistically significant.

## Results

Overall mortality was not statistically different between women (45.2%) and men (48.2%), with 56.6% of patients being female. Age of survivors (52.8 years, 95% C.I. 49.1 –56.5) was not statistically different from non-survivors (46.6 years, 95% C.I. 40.8-52.5, *P*>0.05).There were also no statistically significant association between type of surgery and mortality (Table 1).

**Table 1:** Patient demographics and in-hospital mortality

	**Survivors** **(N=71)**	**Non-Survivors** **(N=75)**	Total cases* **(N=146)**	**P-value**
Age years (95% CI)	52.8 (49.1-56.5)	46.6 (40.8-52.5)	122	NS**
Female Sex	40 (54.8%)	33 (45.2%)	73	NS
Male Sex	29 (51.8%)	27 (48.2%)	56	NS
**Type of Surgery**				NS
Liver	33 (44.0%)	42 (56.0%)	75	
Orthopedic/Spine	12 (44.4%)	15 (55.6%)	27	
Cardiovascular	9 (42.9%)	12 (57.9%)	21	
Obstetric/Gynecologic	8 (88.9%)	1 (11.1%)	9	
Abdominal	4 (80.0%)	1 (20.0%)	5	
Genitourinary	3 (75.0%)	1 (25%)	4	
Thoracic	1 (33.3%)	2 (66.7%)	3	
Neurosurgery	1 (50%)	1 (50%)	2	

Demographic data was assessed for differences in in-hospital mortality. * “Total cases” represents the number of cases reporting this variable. Some variables were not reported in every case report or series from which they emanated. **NS indicates non-significant values.

Changes in EtCO2 as an alerting tool at the time of IOPE were associated with the lowest mortality (30.2%), while patients with PAC changes had a mortality of 53.5%. Although changes in vital signs were the most common presenting sign (60 of 146 cases), this alerting mechanism was also associated with the greatest mortality (72.2%, *P*<0.0001; Table 2). Interestingly, improvements in post-intervention or post-resuscitation vital signs were a statistically significant prognostic factor for survival, irrespective of intervention employed, with 78.1% of patients surviving to hospital discharge (*P*<0.0001). As might be expected, 100% of the patients without improvement in vital signs died, 20.0% of which died in the operating room and the rest within their in-hospital post-operative period. It is worthwhile underscoring that, of the patients with improvement in vital signs post-intervention, 21.9% subsequently died during their hospital stays.

**Table 2:** Monitoring modalities.

**Alerting Tool**	**Survivors (N=65)**	**Non-Survivors (N=75)**	**P-value**
End-tidal Carbon dioxide	30 (69.8%)	13 (30.2%)	<0.0001
Pulmonary Catheter Pressure	20 (46.5%)	23 (53.5%)	<0.0001
Vital Signs Only	15 (27.8%)	39 (72.2%)	<0.0001
**Echocardiographic findings (TTE/TEE)**			
Thrombus Visualization	34 (51.5)	32 (48.5)	0.53
RV Wall Motion Abnormality	43 (61.4)	27 (38.6)	0.003
RV dilatation	24 (70.6)	10 (29.4)	0.003
Tricuspid Regurgitation Jet	11 (84.6)	2 (15.4)	0.007

Monitoring tools used at the time of IOPE were assessed for their association with in-hospital mortality. Six cases were excluded because data for the alerting tool were not reported. New Echocardiographic findings at time of IOPE are also demonstrated and compared between the survivors and non-survivors.

Echocardiographic confirmation of a thrombus was reported in 86.8% of cases reporting whether or not a thrombus was visualized (66 of 76 cases). Of cases that utilized TEE, 92.1% (70 of 76) reported evidence of new right or left myocardial wall motion abnormalities (RLWMA). Right ventricular dilatation (RVD) was reported in 34 of 76 cases reporting TEE use and tricuspid regurgitation (TR) were reported in 13 of 76 such cases. RLWMA, RVD, and TR were each significantly associated with increased mortality, but direct visualization of the thrombus was not (Table 2). Although many authors lauded the utility of TEE as both a diagnostic and a monitoring tool, the pre-event use of this tool did not significantly impact mortality.

**Figure 2:**
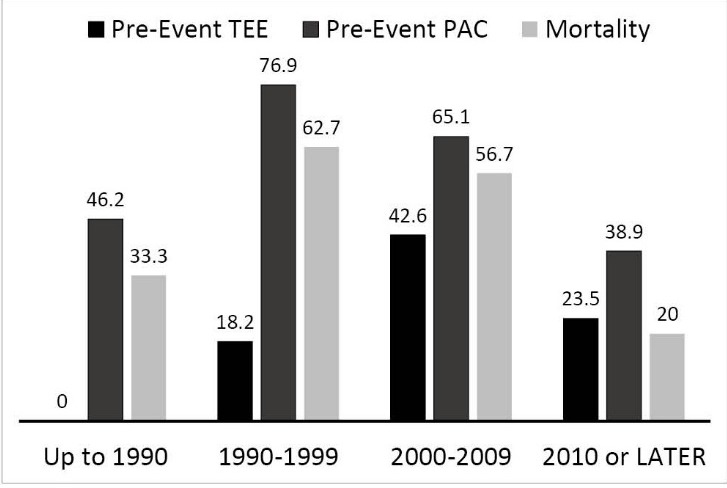
Trends in clinical use of perioperative echocardiography and associated mortality

Clinicians’ trends in resuscitation strategies were assessed comparing cases reported prior to 1990 and for each subsequent decade. Although mortality from massive intraoperative PE’s increased dramatically from the period before 1990 to the decade following 1990, the mortality rate has started to trend down since then (*P*<0.05, Figure 2). The proportion of cases in which echocardiography or pulmonary artery catheters were used to monitor the occurrence of IOPE during these periods does not grossly correlate with mortality trends (Figure 2).

## Discussion

Overall mortality from these events was high at 51.4%, which is consistent with past reports.^3^ Although a rapid deterioration in vital signs appears to be the most common tool alerting practitioners to IOPE events, it is also associated with the greatest mortality (72.2%). This may be because interventions following hemodynamic collapse may be less likely to be successful than interventions initiated with early detection of IOPE (i.e. alert of change in EtCO2; associated with 30.2% mortality). It is also important to recognize that 21.9% of IOPE patients with improvements in vital signs following any interventions or resuscitative measures subsequently died during their hospital stay, suggesting that continued intensive care and close assessment may be warranted for immediate post-IOPE survivors.

One of the hallmarks of this study is the usefulness of EtCO2 in detection of pulmonary embolism in perioperative settings. EtCO2 and its gradient with arterial pressures of carbon dioxide is a known measure of alveolar dead-space,^6^ a primary physiologic marker that is clearly altered during pulmonary embolism.^7-9^ However, the expediency of this tool may be obscured in some patients due to nature of the emboli.^10^ A recent meta-analysis examines the use of capnography as a screening tool of pulmonary embolism.^11^ This meta-analysis examines 14 trials between 1991-2011 that included a total of 2,291 patients with a relatively higher prevalence of pulmonary embolism (20%). These authors calculate a sensitivity of 80% and even a lower specificity of 49% signifying a relatively lower accuracy with large number of false positives, as well as false negatives. Of note in our case series, the observed mortality rate was lower in patients who were diagnosed by means of EtCO2. EtCO2 could additionally provide useful information regarding the state of cardiac output and pulmonary perfusion.^12,13^ This piece of information may be used as a guide to the effectiveness of the resuscitative efforts in patients complicated by cardiac arrest.^14^

Although pre-event placement of a pulmonary catheter added to the diagnostic armamentarium of the perioperative practitioners, the routine use of this invasive monitoring device is not free of risk. There were studies reported in which the patients developed massive *in situ* thrombi using these foreign bodes as the nidus especially in patients with a hypercoagulable state.^15,16^

The pre-event use of the TEE probe was not also associated with an improvement in mortality. However, TEE is commonly believed as a useful diagnostic tool by perioperative physicians.^17,18^ Direct thrombus visualization was possible in 86.8% of cases, allowing for rapid and confirmatory diagnosis of massive IOPE. Unlike findings of RLWMA, RVD, and TR, direct thrombus visualization was not associated with increased mortality. This may be because the RLWMA, RVD, and TR represent direct evidence of myocardial strain, while direct thrombus formation only identifies the source or cause of hemodynamic collapse. The use of TEE for screening or diagnosis of thromboembolic events is not a novel idea and has been used since the early 1990’s in total joint replacement, uniformly revealing embolization following tourniquet deflation.^19^ One possible explanation for this lack of survival benefit from the use pre-event placement of a TEE probe may be due to the concurrent initiation of resuscitation with supportive measures regardless of cause of hemodynamic compromise.

It is important to note that the pathophysiology of thromboembolic events is not identical for all patient populations. While, orthopedic surgery cases are associated with distal thrombus formation from immobilization propagating centrally, massive IOPE’s during liver transplantation surgery (LT) are thought to be the result of different pathophysiology.^1,3,20^ LT’s are performed for severe liver dysfunction, which in itself results in various types of coagulopathy, with a propensity for both bleeding and thrombogenesis.^21-23^ During LT’s, massive PE’s rarely originate from distal venous thrombi. LT’s often present with post-reperfusion syndrome, which often coincides with fresh thrombus formation.^1,22^ In a study designed to screen for emboli using TEE during LT’s, 59% of cases with venovenous bypass had evidence of emboli, compared to 11% in the group without venovenous bypass.^20^ Our review also found that a significant proportion of LT-related PE’s were during the reperfusion phase, reinforcing previous observations.^23^

This study carries the standard limitation of a descriptive, retrospective study (publication, reporting, and recall biases, variable data documentation, and dependence on the quality and limitations of past evidence). Furthermore, due to the wide geographic and temporal distribution of events, resuscitation strategies were not standardized. No effort was made to differentiate between thromboembolic events that were confined to the pulmonary arteries or those that involved a thrombus free-floating in the right heart, yet in patients undergoing LT’s pulmonary emboli alone carry a greater mortality rate than thromboemboli with intra-cardiac components.^3^

In conclusion, this is the first study to formally assess the screening and diagnostic value of various common intraoperative monitoring tools for detection and diagnosis of massive IOPE. Overall mortality was found to be high, consistent with past reports, regardless of tool used. EtCO2 changes as an alerting sign of IOPE were associated with an improved mortality compared to changes in VS as the presenting sign. New findings of RLWMA, RVD, and TR during the IOPE event were associated with greater mortality, likely because these were direct signs of right-sided myocardial strain. Direct thrombus visualization was not associated with worsening mortality, despite providing rapid confirmatory diagnosis of massive IOPE.

## Ethical issues

Not applicable.

## Competing interests

Authors declare no conflict of interest in this study.

## Supplementary materials

Supplementary file 1
contains reference list of case reports and series reviewed.
