# Supplementary material for: Role of Perioperative Monitoring in Diagnosis of Massive Intraoperative Cardiopulmonary Embolism
Source: J Cardiovasc Thorac Res. 2014 Sep 30;6(3):141–5. doi: 10.15171/jcvtr.2014.002 (PMC4195963; doi:10.15171/jcvtr.2014.002)
Supplement: Supplementary file 1 — contains reference list of case reports and series reviewed. [file JCVTR-6-141-s001.docx]

**Role of Perioperative Monitoring in Diagnosis of Massive Intraoperative Cardiopulmonary Embolism**

Ognjen Visnjevac^a^, MD, Leili Pourafkari^b^, MD, Nader D. Nader^a^, MD, PhD, FCCP

**SUPPLEMENTAL DIGITAL CONTENT #1: REFERENCE LIST**

**OF CASE REPORTS AND SERIES REVIEWED**

1. Ellis JE, Lichtor JL, Feinstein SB, Chung MR, Polk SL, Broelsch C, et al.Right heart dysfunction, pulmonary embolism, and paradoxical embolization during liver transplantation. A transesophageal two-dimensional echocardiographic study. Anesth Analg. 1989;68:777-782.
2. Yee LL, Williams GP, Gaither NS, Zurcher RP. Diagnosis of acute intraoperative pulmonary thromboembolism by transesophageal echocardiography. Am Heart J. 1993;125:262-263.
3. Whitby ME, Hellings MJ.Massive intraoperative pulmonary thromboembolism treated by pulmonary embolectomy. Anaesth Intensive Care. 1993;21:342-243.
4. Wenk M, Pöpping DM, Hillyard S, Albers H, Möllmann M.Intraoperative thrombolysis in a patient with cardiopulmonary arrest undergoing caesarean delivery. Anaesth Intensive Care. 2011;39:671-674.
5. Samaan HA.Pulmonary embolism under general anaesthesia, following Esmarch bandage in injuries of lower limb. Anaesthesia. 1970;25:445.
6. Sermeus L, Van Hemelrijck J, Vandommele J, Van Aken H. Pulmonary embolism confirmed by transoesophageal echocardiography. Anaesthesia. 1992;47:28-29.
7. Navalgund AA, Kang Y, Sarner JB, Jahr JS, Gieraerts R.Massive pulmonary thromboembolism during liver transplantation. Anesth Analg. 1988;67:400-402.
8. Cohen JD, Keslin JS, Nili M, Yosipovitch Z, Gassner S.Massive pulmonary embolism and tourniquet deflation. Anesth Analg. 1994;79:583-585.
9. Pharo GH, Andonakakis A, Chandrasekaren K, Amron G, Levitt JD.Survival from catastrophic intraoperative pulmonary embolism. Anesth Analg. 1995;81:188-190.
10. Greilich PE, Randle DW, Froelich EG, Yee LL.Massive intraoperative pulmonary embolism coincident with the administration of succinylcholine. Anesth Analg. 1998;87:491-493.
11. O'Connor CJ, Roozeboom D, Brown R, Tuman KJ.Pulmonary thromboembolism during liver transplantation: possible association with antifibrinolytic drugs and novel treatment options. Anesth Analg. 2000;91:296-299.
12. Fitzsimons MG, Peterfreund RA, Raines DE.Aprotinin administration and pulmonary thromboembolism during orthotopic liver transplantation: report of two cases.Anesth Analg. 2001;92:1418-1421.
13. Planinsic RM, Nicolau-Raducu R, Eghtesad B, Marcos A.Diagnosis and treatment of intracardiac thrombosis during orthotopic liver transplantation. Anesth Analg. 2004;99:353-356
14. Lu CW, Chen YS, Wang MJ.Massive pulmonary embolism after application of an Esmarch bandage. Anesth Analg. 2004;98:1187-1189
15. Lerner AB, Sundar E, Mahmood F, Sarge T, Hanto DW, Panzica PJ.Four cases of cardiopulmonary thromboembolism during liver transplantation without the use of antifibrinolytic drugs. Anesth Analg. 2005;101:1608-1612.
16. Hudcova J, Schumann R.Fatal right ventricular failure with intracardiac thrombus formation during liver transplantation not apparent on postmortem examination. Anesth Analg. 2006;103:506.
17. Schmitz A, Hartmann M. Acute intracardiac thrombus formation during thoracoabdominal aortic surgery. Anesth Analg. 2006;102:1658-1659.
18. Jackson D, Botea A, Gubenko Y, Delphin E, Bennett H. Successful intraoperative use of recombinant tissue plasminogen activator during liver transplantation complicated by massive intracardiac/pulmonary thrombosis.Anesth Analg. 2006;102:724-728.
19. Boone JD, Sherwani SS, Herborn JC, Patel KM, De Wolf AM.The successful use of low-dose recombinant tissue plasminogen activator for treatment of intracardiac/pulmonary thrombosis during liver transplantation. Anesth Analg. 2011;112:319-321.
20. Mangano DT.Immediate hemodynamic and pulmonary changes following pulmonary thromboembolism. Anesthesiology. 1980;52:173-175.
21. Berry AJ.Pulmonary embolism during spinal anesthesia: angiographic diagnosis via a flow-directed pulmonary artery catheter. Anesthesiology. 1982;57:57-59.
22. Pollard BJ, Lovelock HA, Jones RM. Fatal pulmonary embolism secondary to limb exsanguination. Anesthesiology. 1983;58:373-374.
23. San Juan AC Jr, Stanley TH.Pulmonary embolism after tourniquet inflation. Anesth Analg. 1984;63:374.
24. Kang Y, Lewis JH, Navalgund A, Russell MW, Bontempo FA, Niren LS, Starzl TE.Epsilon-aminocaproic acid for treatment of fibrinolysis during liver transplantation. Anesthesiology. 1987 Jun;66(6):766-73.
25. McGrath BJ, Hsia J, Epstein B.Massive pulmonary embolism following tourniquet deflation. Anesthesiology. 1991;74:618-620.
26. Wilson WC, Frankville DD, Maxwell W, Carpenter T, Hastings RH. Massive intraoperative pulmonary embolus diagnosed by transesophageal echocardiography. Anesthesiology. 1994;81:504-508.
27. Sopher M, Braunfeld M, Shackleton C, Busuttil RW, Sangwan S, Csete M.Fatal pulmonary embolism during liver transplantation. Anesthesiology. 1997;87:429-432.
28. Heindel SW, Mill MR, Freid EB, Valley RD, White GC 2nd, Norfleet EA.Fatal thrombosis associated with a hemi-fontan procedure, heparin-protamine reversal, and aprotinin. Anesthesiology. 2001;94:369-371.
29. Fanashawe MP, Shore-Lesserson L, Reich DL.Two cases of fatal thrombosis after aminocaproic acid therapy and deep hypothermic circulatory arrest. Anesthesiology. 2001;95:1525-1527.
30. Nowak NA. Eﬀects of aprotinin and octreotide on cardiopulmonary death rates in liver transplant patients [abstract]. Anesthesiology 2002;96:A170.
31. Szocik J, Rudich S, Csete M.ECMO resuscitation after massive pulmonary embolism during liver transplantation. Anesthesiology. 2002;97:763-764.
32. Wong WH, Braunfeld M, Levin P.Recurrent pulmonary embolism during liver transplantation: possible role of hepatitis B immune globulin as a causative agent. Anesthesiology. 2002;96:1261-1263.
33. Shore-Lesserson L, Reich DL.A case of severe diffuse venous thromboembolism associated with aprotinin and hypothermic circulatory arrest in a cardiac surgical patient with factor V Leiden. Anesthesiology. 2006;105:219-221.
34. Sundt TM 3rd, Kouchoukos NT, Saffitz JE, Murphy SF, Wareing TH, Stahl DJ.Renal dysfunction and intravascular coagulation with aprotinin and hypothermic circulatory arrest. Ann Thorac Surg. 1993;55:1418-1424.
35. Funakoshi Y, Kato M, Kuratani T, Shigemura N, Kaneko M.Successful treatment of massive pulmonary embolism in the 38th week of pregnancy. Ann Thorac Surg. 2004;77:694-695.
36. Jang IS, Kim HT, An SK, Kwon YE, Lee JH. Pulmonary thromboembolism occurred immediately after leg elevation under induction of general anesthesia in a patient with femur fracture: a case report. Anesth Pain Med 2009; 4: 129-132.
37. Boogaerts JG.Lower limb exsanguination and embolism. Acta Anaesthesiol Belg. 1999;50:95-98.
38. Li CH, Lee FJ, Shih YJ, Tsai TC, Peng SK, Luk HN, Huang JH.Massive pulmonary embolism during orthopedic surgery. Acta Anaesthesiol Taiwan. 2007;45:117-120.
39. Sung CS, Chow LH, Sun MS, Yu HP, Ho CM, Tsou MY, Lee TY.Fatal pulmonary embolism in a child undergoing extra-ventricular drainage surgery--a case report. Acta Anaesthesiol Sin. 1999;37:35-39.
40. Araki S, Uchiyama M. Fatal pulmonary embolism following tourniquet inflation. A case report. Acta Orthop Scand. 1991;62:488.
41. Hecker BR, Lynch C. Intraoperative diagnosis and treatment of massive pulmonary embolism complicating surgery on the abdominal aorta. Br J Anaesth. 1983;55:689-691.
42. Goodman NW, Falkner MJ. Massive intraoperative pulmonary embolism in a child. Br J Anaesth. 1987;59:1059-1062.
43. Manji M, Isaac JL,Bion J. Survival from massive intraoperative pulmonary thromboembolism during orthotopic liver transplantation. Br. J. Anaesth. 1998;80:685-687
44. Kottenberg-Assenmacher E, Volbracht L, Jakob H, Greinacher A, Peters J.Disseminated intravascular clotting associated with Fc-receptor IIa-mediated platelet activation in a patient with endocarditis after aortic valve replacement. Br J Anaesth. 2006;97:630-633.
45. Enright AC, Quartey GR, McQueen JD.Pulmonary embolism during operation. Can Anaesth Soc J. 1980;27:65-67.
46. Divekar VM, Kamdar BM, Pansare SN.Pulmonary embolism during anaesthesia: case report. Can Anaesth Soc J. 1981;28:277-279.
47. Woodward DK, Birks RJ, Granger KA.Massive pulmonary embolism in late pregnancy. Can J Anaesth. 1998;45:888-892.
48. Neira VM, Sawchuk C, Bonneville KS, Chu V, Warkentin TE.Case report: management of immediate post-cardiopulmonary bypass massive intra-cardiac thrombosis. Can J Anaesth. 2007:54:461-466.
49. Legare JF, Arora R, Wood JW.Massive intracavitary clot formation during cardiopulmonary bypass. Can J Cardiol. 2004;20:825-826.
50. Cleveland JC, Lebenson IM, Friedman JW.Successful management of massive pulmonary embolism occurring during cardiopulmonary bypass for mitral valve replacement. Chest. 1978;73:236-238.
51. Sisto D, Hoffman D, Camacho M, Fernandes S, Maldarelli W, Lee S.Massive intraoperative pulmonary embolism. Chest. 1992;102:307-308.
52. Theuerkauf I, Harbrecht U, Pütz U, Fischer HP.Massive pulmonary capillary occlusion by microthrombi. Unexpected cause of fatal right heart failure during liver transplantation. Chirurg. 2002;73:380-382.
53. Martins PN, Kim-Schluger L, Rodriguez-Davalos M, Martins AB, Krachkova N, Facciuto M, Sheiner P. Massive pulmonary and intracardiac embolism during liver transplantation. Exp Clin Transplant. 2010;8:184-188.
54. Jha NK, Rezk AI, Omran AS, Hussain A, Najm HK. Acute pulmonary thromboembolism during mitral valve repair. Heart Lung Circ. 2008;17:159-161.
55. Shi XY, Xu ZD, Xu HT, Jiang JJ, Liu G.Cardiac arrest after graft reperfusion during liver transplantation. Hepatobiliary Pancreat Dis Int. 2006;5:185-189.
56. Farrell FJ, Nguyen M, Woodley S, Imperial JC, Garcia-Kennedy R, Man K, et al.Outcome of liver transplantation in patients with hemochromatosis. Hepatology. 1994;20:404-410.
57. Darmanis S, Papanikolaou A, Pavlakis D.Fatal intra-operative pulmonary embolism following application of an Esmarch bandage. Injury. 2002;33:761-764.
58. Simek M, Nemec P, Cermák M, Prikrylová K.Intraoperative massive pulmonary embolism during coronary artery bypass grafting. Interact Cardiovasc Thorac Surg. 2005;4:283-284.
59. Hofmann AA, Wyatt RW. Fatal pulmonary embolism following tourniquet inflation. A case report. J Bone Joint Surg Am. 1985;67:633-634.
60. Lobato EB, Seubert CN, Bott J, Knauf DG. Cardiac arrest after uncomplicated right upper lobectomy. J Cardiothorac Vasc Anesth. 1999;13:105-106.
61. Lichtman AD, Carullo V, Minhaj M, Karkouti K.Case 6--2007: massive intraoperative thrombosis and death after recombinant activated factor VII administration. J Cardiothorac Vasc Anesth. 2007;21:897-902.
62. Mejia A, Mendoza ML, Kieta D, Gulden H, Aramoonie AE, Lee GW, Cheng S.Nonfatal intracardiac thromboembolism during liver transplantation. J Cardiothorac Vasc Anesth. 2010;24:109-111.
63. Nicoara A, Assaad S, Geirsson A, Rousou A, Jadbabaie F.Unexpected intraoperative diagnosis of pulmonary embolism by transesophageal echocardiography. J Cardiothorac Vasc Anesth. 2010;24:639-640.
64. Shillcutt SK, Brakke TR, Montzingo CR, Agrawal A.Intraoperative diagnosis of acute pulmonary embolus by transesophageal echocardiogram. J Cardiothorac Vasc Anesth. 2011;25:603.
65. Jorgenson A, Jaeger JM, de Souza DG, Blank RS. Acute intraoperative pulmonary embolism: an unusual cause of hypoxemia during one-lung ventilation. J Cardiothorac Vasc Anesth. 2011;25:1113-1115.
66. Obray JB, Long TR, Rehfeldt KH, Wass CT.Cardiovascular collapse during elective orthopedic surgery: massive intraoperative pulmonary thromboembolism treated with emergent cardiopulmonary bypass. J Clin Anesth. 2005;17:205-208.
67. Pivalizza EG, Ekpenyong UU, Sheinbaum R, Warters RD, Estrera AL, Saggi BH, Mieles LA.Very early intraoperative cardiac thromboembolism during liver transplantation. J Cardiothorac Vasc Anesth. 2006;20:232-235.
68. Ellenberger C, Mentha G, Giostra E, Licker M.Cardiovascular collapse due to massive pulmonary thromboembolism during orthotopic liver transplantation. J Clin Anesth. 2006;18:367-371.
69. Rajan GR.Intractable intraoperative hypoxemia secondary to pulmonary embolism in the presence of undiagnosed patent foramen ovale. J Clin Anesth. 2007;19:374-377.
70. Lee CL, Ong J, Chang BS, Chen TY, Lai HY. Accidental pulmonary emboli noted by TEE during aortic valve replacement: a case report. J Clin Anesth. 2011;23:231-233.
71. Ayad S, Tetzlaff JE.Massive pulmonary embolism in a patient undergoing Cesarean delivery. J Clin Anesth. 2012;24:582-585.
72. Sankey EA, Crow J, Mallett SV, Alcock RJ, More L, Burroughs AK, Rolles K.Pulmonary platelet aggregates: possible cause of sudden peroperative death in adults undergoing liver transplantation. J Clin Pathol. 1993;46:222-227.
73. Alvarez JM, Goldstein J, Mezzatesta J, Flanagan B, Dodd M.Fatal intraoperative pulmonary thrombosis after graft replacement of an aneurysm of the arch and descending aorta in association with deep hypothermic circulatory arrest and aprotinin therapy. J Thorac Cardiovasc Surg. 1998;115:723-724.
74. Estrera AS, King RP, Platt MR.Massive pulmonary embolism: a complication of the technique of tourniquet ischemia. J Trauma. 1982;22:60-62.
75. Lee JY, Lee SY, Shin I, Park C, Lee BS, Kim MS.Fatal pulmonary embolism and coincidental cerebral infarction after spinal anesthesia -A case report-. Korean J Anesthesiol. 2011;61:515-518.
76. Song JE, Chun DH, Shin JH, Park C, Lee JY.Pulmonary thromboembolism after tourniquet inflation under spinal anesthesia -A case report-. Korean J Anesthesiol. 2010;59:S82-S85.
77. Oda K, Sato N, Ishii H, Agatsuma T, Hashimoto K, Sato S. Acute massive pulmonary embolism occurring during orthopedic surgery. Kyobu Geka. 2003;56:356-359.
78. Gologorsky E, De Wolf AM, Scott V, Aggarwal S, Dishart M, Kang Y. Intracardiac thrombus formation and pulmonary thromboembolism immediately after graft reperfusion in 7 patients undergoing liver transplantation. Liver Transpl. 2001;7:783-789.
79. Ramsay MA, Randall HB, Burton EC.Intravascular thrombosis and thromboembolism during liver transplantation: antifibrinolytic therapy implicated? Liver Transpl. 2004;10:310-314.
80. Gosseye S, van Obbergh L, Weynand B, Scheiff JM, Moulin D, de Ville de Goyet J, Otte JB.Platelet aggregates in small lung vessels and death during liver transplantation. Lancet. 1991;338:532-534.
81. Wang A, Patel R. Massive Intracardiac/pulmonary migrating thromboembolism and gastric variceal hemorrhage during orthotopic liver transplantation [abstract].Proceedings of the 2001 Western Anesthesia Research Conference.
82. Strickland J, Ahmed K, Ayoub T, Patel R. Intra-cardiac thrombosis during liver transplant [abstract]. Proceedings of the 2003 Western Anesthesia Research Conference.
83. Suriani RJ, Cutrone A, Cohen E, Konstadt SN, Gabrielseon GV, Miller C. Pulmonary thromboembolism during liver transplantation: Is venovenous bypass protective [abstract]? Liver Transpl Surg 1995; 1: 416A
84. De Wolf A, Gologorsky E, Scott V, Aggarwal S, Dishart M, Kang Y. Intravascular and intracardiac thrombous formation and pulmonary thromboembolism immediately after graft reperfusion in six patients undergoing liver transplantation[abstract]. Liver Transpl Surg 1995; 1: 416A
85. Kato S, Okada K, Sakuramoto C, Okutomi T, Takenaka T, Goto F. Fatal pulmonary embolism during knee surgery under epidural anesthesia. Masui. 1997;46:393-396.
86. Tsubota S, Watanabe T, Hamaura M, Miyamoto Y.A case of pulmonary embolism associated with pneumatic tourniquet deflation. Masui. 2001;50:293-295.
87. Porhomayon J, Perala PR, Purandath L, Annam S, Nader ND. Massive Intraoperative Pulmonary Emboli Following Thrombectomy of Hemodialysis Fistula. Sphere. 2011;63:41-46
88. Baubillier E, Cherqui D, Dominique C, Khalil M, Bonnet F, Fagniez PL, Duvaldestin P.A fatal thrombotic complication during liver transplantation after aprotinin administration. Transplantation. 1994;57:1664-1666.
89. Prah GN, Lisman SR, Maslow AD, Freeman RB, Rohrer RJ.Transesophageal echocardiography reveals an unusual cause of hemodynamic collapse during orthotopic liver transplantation--two case reports. Transplantation. 1995;59:921-925.
90. Kazanci D, Turan S, Bostanci B, Ünver S, Aydinli B, Erdemli Ö, Özgök A, Akoğlu M, Ulus AT.Cardiac arrest that developed during liver transplantation and intervention with cardiopulmonary bypass: case report. Transplant Proc. 2012;44:1759-1760.
91. Lash AE, Choyke PL, Walther MM.Rapid extension of vena caval thrombus. Tech Urol. 1996;2:207-209.
